# Dendritic Cell Cross-Priming Is Essential for Immune Responses to *Listeria monocytogenes*

**DOI:** 10.1371/journal.pone.0007210

**Published:** 2009-10-06

**Authors:** Anna T. Reinicke, Kyla D. Omilusik, Genc Basha, Wilfred A. Jefferies

**Affiliations:** The Biomedical Research Centre, Michael Smith Laboratories, Departments of Medical Genetics, Microbiology and Immunology, and Zoology, The University of British Columbia, Vancouver, British Columbia, Canada; Karolinska Institutet, Sweden

## Abstract

Cross-presentation is now recognized as a major mechanism for initiating CD8 T cell responses to virus and tumor antigens *in vivo*. It provides an elegant mechanism that allows relatively few Dendritic cells (DCs) to initiate primary immune responses while avoiding the consumptive nature of pathogenic infection. CD8 T cells play a major role in anti-bacterial immune responses; however, the contribution of cross-presentation for priming CD8 T cell responses to bacteria, *in vivo*, is not well established. *Listeria monocytogenes* (*Listeria*) is the causative agent of Listeriosis, an opportunistic food-borne bacterial infection that poses a significant public health risk. Here, we employ a transgenic mouse model in which cross-presentation is uniquely inactivated, to investigate cross-priming during primary *Listeria* infection. We show that cross-priming deficient mice are severely compromised in their ability to generate antigen-specific T cells to stimulate MHC I-restricted CTL responses following *Listeria* infection. The defect in generation of *Listeria*-elicited CD8 T cell responses is also apparent *in vitro*. However, in this setting, the endogenous route of processing *Listeria*-derived antigens is predominant. This reveals a new experimental dichotomy whereby functional sampling of *Listeria*-derived antigens *in vivo* but not *in vitro* is dependent on cross-presentation of exogenously derived antigen. Thus, under normal physiological circumstances, cross-presentation is demonstrated to play an essential role in priming CD8 T cell responses to bacteria.

## Introduction

Antigen processing and subsequent presentation to specific T cells is necessary for effective bacterial immune responses involving both CD4 and CD8 T cells. While CD4 helper T cell responses are necessary for limiting infection, CD8 cytotoxic T lymphocytes are required for clearance of bacteria [1]. MHC class-II antigen presentation is mainly restricted to internalized proteins that are processed and loaded in phagosomal/endosomal compartments for activation of CD4 T helper cells [2]. MHC I presentation is classically restricted to endogenously synthesized antigens of viral or self origin [3]. Such proteins are digested by the proteasome and translocated into the endoplasmic reticulum (ER) via the transporters associated with antigen processing, TAP1 and TAP2 for loading on MHC I. A second, less well-defined mechanism of processing has also been described and is referred to as cross-presentation whereby exogenously-derived antigens may be captured from other sources and following uptake are processed in such a way to access MHC I molecules for loading and presentation [4].

There are several mechanisms proposed to explain how exogenously-derived antigens that have been phagocytosed or macropinocytosed may be processed and loaded on MHC I molecules for presentation. In one model, exogenous antigens are proposed to be processed directly in a compartment within the endocytic pathway in a TAP and proteosome independent manner where they are degraded by proteases and cathepsins for loading [5]–[7]. In a second model, internalized antigens may be delivered to the cytosol where they can follow the classical proteosome-mediated degradation and entry to the ER via TAP for loading on nascent MHC I molecules [8], [9]. Membrane of the ER has been proposed to fuse with the phagosome during phagocytosis [10] that would allow the delivery of antigens into the cytosol. In addition, ER phagosome fusion has also led to studies showing that phagosomes are competent organelles for proteosome and TAP dependent cross-presentation [11]–[13]. Thus, the mechanisms proposed are non-mutually exclusive and the importance of one or other may depend on the source of antigen.

Many antigens have been shown to be cross presented *in vitro* including soluble proteins, cellular antigens, immune complexes, intracellular bacteria and parasites [14]. Indeed, the primary significance of cross-presentation is shown *in vivo* as a major mechanism for initiating CD8 T cell priming [5], [15]. A key role of cross-priming *in vivo* has been demonstrated to initiate CTL immunity during virus infection in a system where only non-hematopoetic cells were infected and bone marrow-derived APCs were shown to be required to capture viral antigens from infected cells by cross-priming [16].

Cross-presentation of bacterial antigens on MHC I has been demonstrated *in vitro* [17] and several groups have provided data for a mechanism whereby apoptosis of infected cells would provide the source of antigen and result in uptake of bacteria-encoded antigens by bystander DCs which have the capacity to activate naïve T cell responses [18]–[20]. *In vivo*, pathogen-infected APCs may be functionally compromised and DCs may not be directly infected. Thus, we pose that an exogenous pathway for processing bacterial-derived antigens to prime CD8 T cell responses would indeed be required; however, this has not been directly addressed. The contribution of cross-priming versus direct priming in immunity to such intracellular pathogens *in vivo* is therefore not well established owing to difficulties associated with distinguishing these mechanisms of initiating cellular immune responses.

Recent data from our laboratory demonstrated that MHC I trafficking is controlled by a highly conserved tyrosine located in the cytoplasmic tail of the class I molecule. Transgenic mice expressing either the wild type H-2K^b^ MHC I allele (K^b^WT), or H-2K^b^ containing a single point mutation substituting a phenylalanine residue for the conserved tyrosine in exon 6 of the molecule (ΔY) were generated and bred onto a H-2^K^ background by backcrossing transgenic founders with C3H/He mice. Our results showed that the highly conserved MHC I cytoplasmic tyrosine residue forms part of an intracellular targeting motif that is required for routing of MHC I molecules through endolysosomal compartments in DCs where MHC I loading of cross-presented exogenous peptides occurs [5]. The functional consequences of the cytoplasmic tail mutation were demonstrated for two anti-viral CTL responses *in vivo* whereby the tyrosine mutation abrogated cross-priming of those viral epitopes. Here, we are using this transgenic model system to address the contribution of cross-priming during intracellular bacterial infection.

*Listeria monocytogenes* (*Listeria*) is a gram-positive facultative intracellular bacterial pathogen. It is the causative agent of listeriosis, a food borne disease characterized by systemic infection of *Listeria* disseminating from the intestine into the blood stream and organs. Listeriosis is the leading cause of death among pathogenic bacteria. Pregnant women, neonates, and elderly or immunocompromised individuals are particularly at risk from infection but apparently healthy individuals may also be affected. Listeriosis is normally sporadic with few cases per year but outbreaks of epidemic proportions still occur. Following entry into the host cell *Listeria* expresses a virulence factor, Listeriolysin O (LLO). This lyses the phagosomal membrane and allows the bacteria to escape the phagosome into the cytosol where it survives and replicates. *Listeria* infection can spread from cell to cell without entering the extracellular domain and therefore a functional CD8 T cell response is required for clearance of the bacteria and development of protective immunity [21]–[23]. *Listeria*-derived MHC I- restricted peptides may be generated from proteins secreted into the cytosol such as the virulence determinant, LLO [24]. Thus, secreted bacterial proteins may be directed through the classical endogenous route of processing for presentation on MHC I. However, following infection *in vivo*, bacteria are found preferentially within the cytosol of macrophages and hepatocytes in the spleen and liver [25]. Therefore, professional antigen presenting cells (pAPCs) such as DCs may internalize antigens from infected cells or bacterially-derived apoptotic debris for initiation of CD8 T cell responses.

DCs are now considered essential for priming of naïve CD8 T cell responses owing to their unique maturation and migration ability, high expression of co-stimulatory molecules, and unique pH sensing and NADPH oxidase 2 (NOX2) activity [26]. Indeed, DCs are extraordinarily well adapted to cross present antigens to CD8 T cells most efficiently [5], [27]. Further, DCs have been shown to be essential in immune responses to bacterial pathogens since they are critical in priming CD8 T cells to bacterial pathogens. In one study, a diphtheria toxin-based system was used to induce short-term ablation of DCs and showed that the anti-*Listeria* CTL response was critically dependent on DCs [28]. In a second study, Lenz et al. showed that the priming of CTL to *Listeria* is restricted to bm-derived APCs [29]. Thus, DC cross-priming of CD8 T cells may provide an important means of alerting cellular immune responses to *Listeria monocytogenes* bacterial infection. Here, we directly examine the hypothesis that cross-presentation is essential for initiating CD8 T cell responses and mount anti-bacterial responses *in vivo*.

## Results

### The strength of an antigen specific CD8 T cell response following *Listeria*-OVA infection *in vivo* differs depending on the route of infection

**Figure 1:**
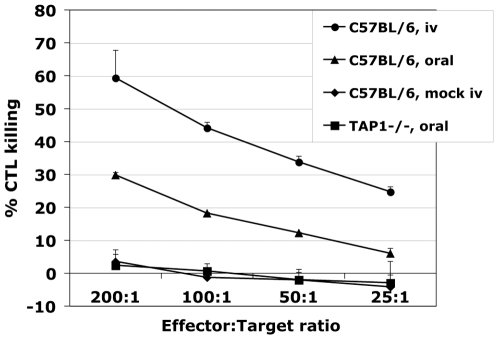
Antigen specific CD8 T cell responses in C57BL/6 mice differ following *Listeria*-OVA oral and i.v. routes of infection. C57BL/6 mice were infected with 1e^9^ cfu or 1e^4^ cfu *Listeria*-OVA by oral gavage or i.v. injection, respectively. Mock oral infections were performed with 0.1 M HEPES-PBS (Mock) while PBS alone was used for mock i.v. infections. At the peak of the cellular response, 9 days post infection, spleens were harvested and the cytolytic capacity of CD8 T cells was analysed in a standard ^51^-Cr release assay. T cells were expanded in culture with OVA_(257–264)-_specific peptide for 6 days. Effector T cells were then incubated with OVA_(257–264)_ peptide pulsed, sodium chromate-labeled target cells and their ability to lyse targets was measured at indicated ratios. Experiments were performed in triplicate with 3 mice per group.

Following *Listeria* infection, bacteria are preferentially found within the cytosol of macrophages and hepatocytes of the spleen and liver [25]. Bacteria spread from cell to cell without leaving the intracellular compartment, thereby making CD8 T cell activation crucial for clearance of the bacteria and induction of protective immunity [30]. The route of administration of bacteria may greatly affect the strength and type of adaptive immune response generated. In particular, the contribution of cross-presentation may be influenced. Here, we examined primary CD8 T cell responses following i.v. and oral gavage infection of C57BL/6 mice to establish a model examining the initiation of adaptive immunity to *Listeria*. In order to analyse Ag-specific CD8 T cell responses to *Listeria* infection, a recombinant *Listeria* strain (*Listeria*-OVA) that expresses a well-defined model antigen, ovalbumin (OVA), was used. Specific T cell responses were measured by CTL killing assays of spleen preparations taken 9 days post infection and expanded in culture by *in vitro* boosting with MHC I immunodominant OVA_(257–264)_ peptide. The ability of activated CD8 T cells to kill target cells presenting OVA_(257–264)_ peptide in the context of H-2K^b^ was measured. Splenic CTLs from i.v. infection were found to have more robust killing capacity compared to those generated following oral infection (Fig. 1). However, specific responses could be measured following oral infection thereby allowing us to assess the contribution of cross-priming in an oral bacterial infection model, which is the natural route of infection of this bacterium.

### ΔY mice are deficient in generating *Listeria*-derived specific CD8 T cell responses *in vivo*

To gain insight into the role of cross-presentation for priming T cell responses following bacterial infection, we evaluated CD8 T cell responses following *Listeria*-OVA infection in the H-2K^b^ transgenic models. Cross-presentation is uniquely inactivated in the ΔY mice thereby allowing the contribution of cross-priming during oral infection to be tested specifically. Interestingly, transgenic mice showed increased susceptibility to *Listeria*-OVA infection and thus lower infection doses were administered for sub-lethal infection.

**Figure 2:**
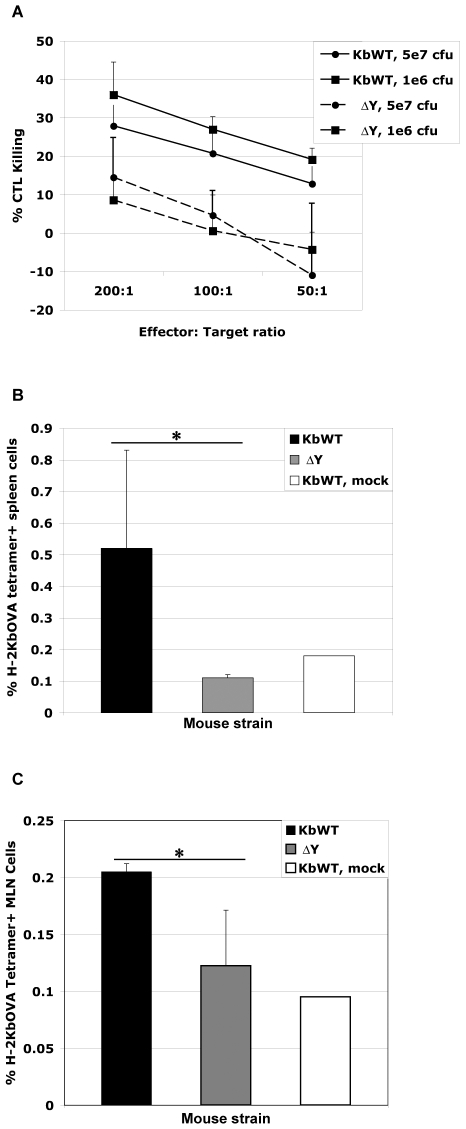
ΔY mice are deficient in generating specific CD8 T cell responses following *Listeria*-OVA infection. K^b^WT and ΔY mice were orally infected with 5e^7^ or 1e^6^ cfu *Listeria*-OVA or mock infected with 0.1 M HEPES-PBS. Spleens and MLN were harvested 9 days post infection and analysed directly *ex vivo* or specific T cells were expanded in culture by incubation with OVA_(257–264)_ peptide A, Cytotoxicity assays were performed whereby OVA_(257–264)_ peptide pulsed, sodium chromate-labeled target cells were incubated with restimulated effector T cells from spleens of infected mice. The ability of effector T cells to lyse targets was measured as chromium release at indicated ratios. 2 mice per group were analysed for each dose of infection tested. Cytotoxicity experiments were performed in triplicate. B, C, *Listeria*-derived OVA_(257–264)_-specific CD8 T cells from spleens or MLN of 2 mice per group were quantified by tetramer staining following oral infection with 5e^7^ cfu *Listeria*-OVA and restimulation of the T cells *in vitro* (B) or analysed directly *ex vivo* (C). Results are presented as bar graphs of fold increases in % tetramer/CD8 T cells stained plus SD; Student's *t* test *, *p*<0.05.

CTL killing assays were performed to test the efficiency of activated T cells from infected spleens of transgenic mice to recognize and lyse target cells. Equal numbers of splenic CTLs from K^b^WT and ΔY were used in the assays and differed significantly in their ability to kill peptide-pulsed targets at infection doses of 5e7 cfu (*p<0.05*) and 1e6 cfu (*p<0.005*) tested (Fig. 2A). Quantification of *Listeria*-specific CD8 T cells was performed on spleen and draining mesenteric lymph nodes (MLN) from K^b^WT and ΔY mice following oral gavage infection. Splenocytes were re-stimulated *in vitro* with MHC I immunodominant OVA_(257–264)_ peptide and tetramer staining performed to detect H-2K^b^OVA_(257–264)_ specific CD8 T cells. ΔY mice had significantly lower percentage of H-2K^b^OVA_(257–264)_ CD8 T cells in spleen (Fig. 2B) indicating that ΔY mice are deficient in developing antigen specific CD8 T cell responses to *Listeria* infection. Quantification of *Listeria-*OVA-specific CD8 T cells was also performed on MLN directly *ex vivo* following oral gavage infection transgenic mice. Again, ΔY mice generated significantly fewer H-2K^b^ OVA_(257–264)_-specific CD8 T cells following *Listeria*-OVA infection (Fig. 2C). The results indicate that antigen-specific CD8 T cells from ΔY mice are deficient in developing cytotoxic T cell responses following infection.

**Figure 3:**
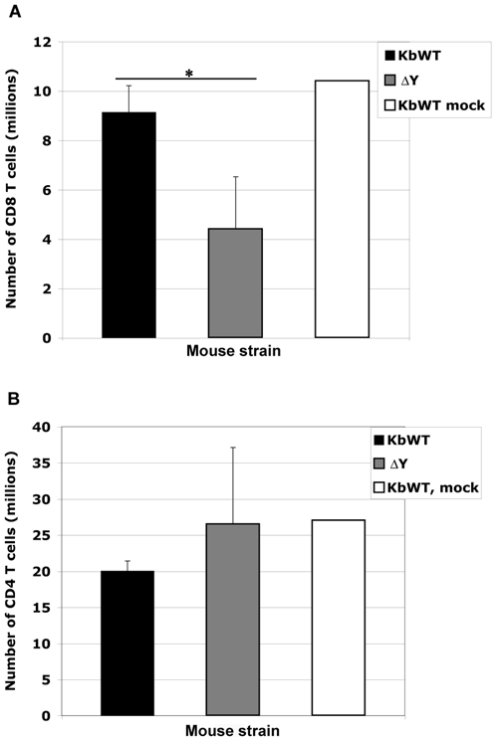
Depletion of CD8 T cells in ΔY spleen following *Listeria*-OVA infection. K^b^WT and ΔY mice were orally infected with 5e^7^ cfu *Listeria*-OVA or mock infected with Hepes buffered PBS. Infected spleens were harvested 9 days post infection and OVA-specific T cells expanded in culture for 5 days by incubation with specific OVA_(257–264)_ peptide. Cells were then counted by trypan blue exclusion and CD8 and CD4 T cells were stained and analysed by flow cytometry. Numbers of T cells were calculated and are presented as a bar graph plus SD of 2 mice per group infected; student's *t* test *, *p*<0.05.

Notably, the percentage of total CD8 T cells generated in spleen following *Listeria* infection was observed lower in ΔY spleen compared to mock, K^b^WT or C57BL/6 infections. Indeed, T cell depletion following *Listeria* infection has been reported but only in the first 48 h of infection [31], [32]. To examine whether *Listeria* bacterial infection resulted in significant depletion of T cells in spleen, 9 days post infection, analysis of CD8 T cell numbers following *in vitro* stimulation was carried out. The numbers of CD8 T cells were significantly lower in ΔY mice compared to K^b^WT while the numbers of CD4 T cells did not significantly differ (Fig. 3A, B).

### ΔY DCs are deficient in stimulating CD8 T cell responses following *Listeria* infection *in vitro*

Dendritic cells from K^b^WT and ΔY mice are used to extend the original findings that the highly conserved tyrosine residue in the cytoplasmic domain of MHC I is responsible for endolysosomal trafficking in DCs thereby allowing processing of exogenously-derived antigen for cross-presentation [5]. To address the function of H-2K^b^ molecular trafficking on antigen processing for presentation and activation of T cell responses, we evaluated T cell activation and proliferation following *Listeria*-OVA infection of DCs from transgenic mice *in vitro*.

Immature DCs were generated from bone marrow (bmDCs) of transgenic K^b^WT and ΔY mice by 10 day culture with GM-CSF. DCs from wild type and mutant transgenic mice both express equivalent amounts of the DC-specific marker, CD11c, MHC I and class II molecules on their surface (data not shown).

**Figure 4:**
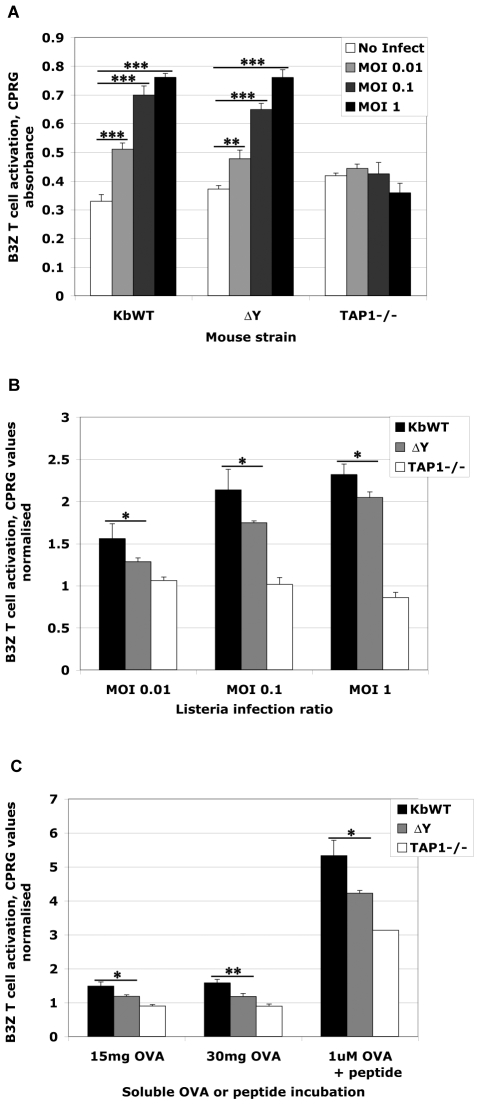
ΔY DCs have decreased ability to activate T cells following *in vitro* infection with *Listeria*-OVA. Bone marrow preparations from K^b^WT and ΔY mice were cultured *in vitro* with GM-CSF to develop bmDCs. After 10 days in culture, cells were labeled with CD11c and H-2K^b^ and analysed by flow cytometry. A, Day 10 bmDCs from K^b^WT, ΔY or TAP1^−/−^ mice were infected with increasing doses of *Listeria*-OVA for 4 h. After 4 h infection time, extracellular bacteria were removed and DCs were subsequently incubated overnight to allow processing of internalized bacteria. DCs were then incubated with the B3Z T cell hybridoma for 18 h and T cell activation was measured using a CPRG chemiluminescent assay. Uninfected bmDCs from of K^b^WT, ΔY or TAP1^−/−^ mice served as negative controls. B, Data is presented as a fold increase in T cell activation by dividing CPRG absorbance values by background C. uninfected bmDCs from K^b^WT, ΔY or TAP1^−/−^ mice were incubated with indicated doses of soluble whole OVA. Processing of internalized OVA was allowed to take place overnight followed by incubation with B3Z T cell hybridoma for 18 h before measuring T cell activation. BmDCs were also pulsed for 1 h with 1 µM OVA_(257–264)-_specific peptide as positive control. Experiments were performed in triplicate and values are presented as means plus SD with Student's *t* tests * *p*<0.05; ** *p*<0.005; ***p<0.0005.

DCs were infected with *Listeria*-OVA at MOIs, as indicated. Infections were terminated after 4 h by washing to remove extracellular bacteria and resuspending the cells in media with 10 µg/ml chloramphenicol to kill any remaining extracellular bacteria. DCs were allowed to process and present endogenously-derived (from direct infection) and any remaining exogenously-derived (from dead bacteria) bacterial antigens overnight. After 18 h, DCs were incubated with B3Z T cells, a T cell hybridoma that is activated by the recognition of H-2K^b^ in association with OVA_(257–264)_ peptide. K^b^WT and ΔY DCs infected with *Listeria*-OVA were equally adept to process *Listeria*-derived antigens to activate B3Z T cells in a dose-dependent manner (p<0.005, p<0.0005) while infected TAP1−/− DCs were unable to activate B3Z (Fig. 4A). Interestingly, ΔY DCs showed reduced ability to activate B3Z T cells compared to K^b^WT DCs when differences in background were taken into account (Fig. 4b), although the statistical significance was smaller (p<0.05) compared to the dose dependant activation found for both K^b^WT and ΔY DCs, indicating a smaller contribution of cross-presentation, probably from extracellular dead bacteria, compared to direct presentation *in vitro*. In addition, cross-presentation of soluble whole OVA by K^b^WT but not ΔY DCs was observed as had been previously shown (Fig. 4C).

### ΔY APCs are deficient in stimulating naïve T cell proliferation following *Listeria*-OVA infection

**Figure 5:**
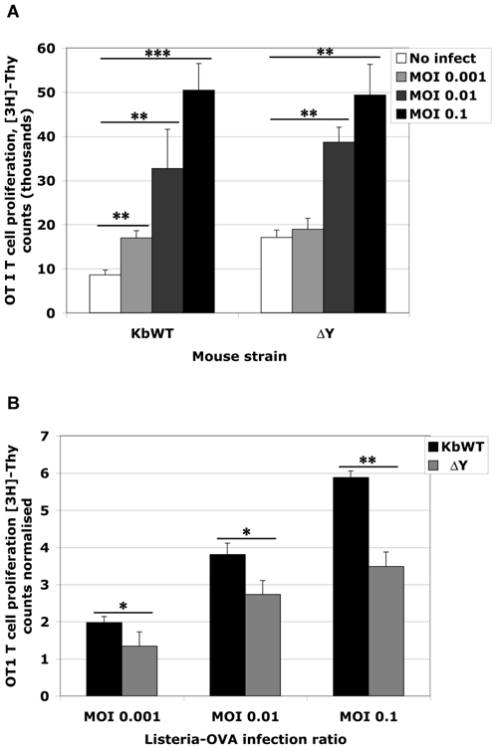
ΔY APCs have decreased ability to stimulate naive T cell proliferation following *in vitro* infection with *Listeria*-OVA. A. Splenocytes from K^b^WT and ΔY mice were infected with increasing doses of *Listeria*-OVA. Following 4 h infection, extracellular bacteria were removed and infected cells were incubated overnight. APCs were then cultured with OT-I T cells for 48 h. [^3^H]-Thymidine was then added to the cultures and 24 h later, proliferation was measured by [^3^H]-Thy incorporation. B. Data is presented as fold increases in T cell proliferation. Values are calculated by dividing [3H]-Thy count values by background. Experiments were performed in triplicate and values are presented as means plus SD with Student's *t* tests * *p*<0.05; ** *p*<0.005; ***p<0.0005.

Antigen processing and presentation to stimulate naïve T cells was next tested. For this, freshly isolated splenocytes were counted and infected with *Listeria*-OVA for 4 h before washing and resuspending cells in media plus antibiotics to terminate the infection. Eighteen hours later, cells were incubated with naïve CD8 OT-I T cells which recognize APCs presenting OVA_(257–264)_ peptide in the context of H-2K^b^ molecules. ^3^H-[Thy] incorporation by proliferating OT-I in response to activation was measured 48 h later. Similarly to what was found in bmDC infections, both K^b^WT and ΔY splenocytes processed and presented *Listeria*-derived antigen to stimulate naïve CD8 T cell proliferation in a dose dependant manner (p<0.005, p<0.0005) (Fig. 5A). Significant differences between ΔY and K^b^WT splenocytes to activate OT-I T cells were also observed (p<0.05, p<0.005) (Fig. 5B) indicating the role of cross-presentation for processing and presenting *Listeria*-OVA antigens.

### Loading and Presentation of *Listeria*-OVA-derived antigens on ΔY and K^b^WT APCs

**Figure 6:**
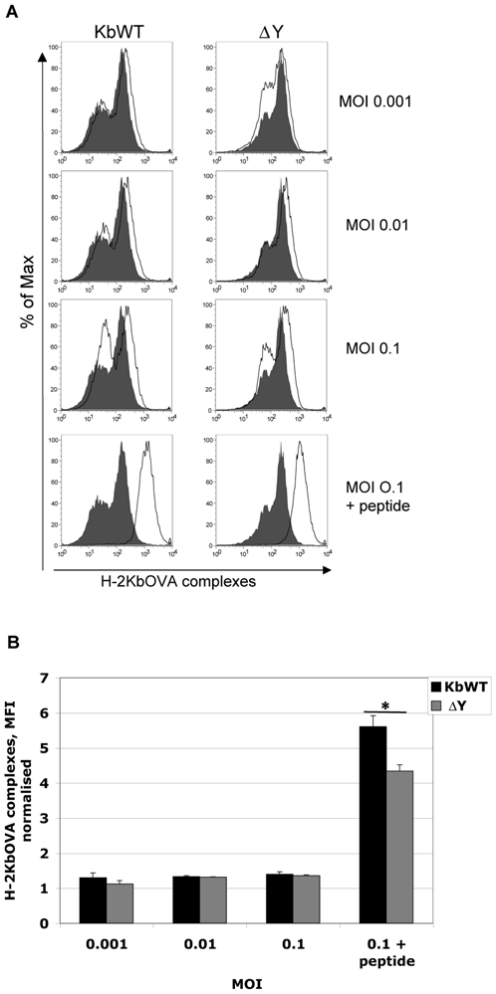
Exchange of H-2K^b^OVA peptide is affected by MHC I tyrosine mutation on ΔY APCs. Splenocytes from K^b^WT and ΔY mice were infected with *Listeria*-OVA between 0.001–0.1 MOI. Following 4 h infection, extracellular bacteria were removed and infected cells were incubated overnight. A. APCs were surface stained with a monoclonal antibody (mAb 25.D1.16) that specifically recognizes H-2K^b^-OVA_(257–264)_ complexes and analysed by flow cytometry. Uninfected APCs or infected APCs pulsed with 1 µM OVA_(257–264)_ peptide for 1 h were used as negative and positive controls, respectively. B. Data is represented as a bar graph of the mean fluorescence intensity (MFI) of surface H-2K^b^OVA_(257–264)_ complexes. Experiments were performed in triplicate and values are presented as average MFI plus SD with Student's *t* tests * *p*<0.05.

*In vitro* infection of DCs and APCs indicated that the deficiency observed in ΔY mice to generate CD8 T cell responses against *Listeria*-OVA lies in its APCs, which are defective in stimulating fully functioning T cells. To address the mechanism of the defect, the antigen processing and loading capacity following *Listeria*-OVA infection was investigated in ΔY and K^b^WT splenocytes. Following 4 h infection with *Listeria*-OVA, splenocytes were incubated overnight and surface stained with the mAb 25.D1.16 which specifically recognizes H-2K^b^-OVA_(257–264)_ complexes. Both ΔY and K^b^WT splenocytes showed loading and presentation of *Listeria*-OVA derived antigen (Fig. 6A). Marginal differences in loading and presentation were observed between ΔY and K^b^WT splenocytes at MOI 0.001 and MOI 0.1 but were not significant by students' t test (Fig. 6B). Interestingly, infected ΔY splenocytes pulsed with OVA_(257–264)_ peptide resulted in significantly less presentation of H-2K^b^-OVA_(257–264)_ complexes (Fig. 6B). Expression of H-2K^b^ molecules is normally similar or higher on ΔY APCs compared to K^b^WT APCs [6]. The latter result indicates that aberrant recycling of MHC I molecules on ΔY APCs which has been shown previously [6], may contribute to the decreased overall levels of surface H-2K^b^-OVA_(257–264)_ complexes following infection and peptide pulsing.

## Discussion

The physiological relevance of cross-presentation *in vivo* has been related to the need to generate CTL immunity to virus-infected cells and tissue-specific viruses [5], [16] or following major virus and tumor escape mechanisms resulting in down-regulation of antigen presentation [33], [34]. Cross-presentation is now established under various investigational conditions as a fundamental pathway of activating CD8 T cells but understanding its significance for mounting immune responses *in vivo* has been limited by experimental means to distinguish cross-priming from overall MHC I priming. Here, we have taken advantage of a transgenic mouse model in which a tyrosine motif in the cytoplasmic tail of MHC I has been mutated. This mutation abrogates the targeting of MHC I molecules to endolysosomal compartments where they may undergo peptide exchange and acquire exogenously derived antigens for cross-presentation [5], [6]. In this context, we have used the mouse model to examine the role of cross-presentation of bacterially-derived antigens following *Listeria* infection *in vivo*.

During naturally acquired infection, bacteria traverse the epithelial-cell layer and disseminate in the bloodstream to other organs, such as the spleen and liver, [30]. We show that the ability to generate *Listeria*-specific CD8 T cells with cytotoxic capacity is affected by the route of infection. Systemic infection induced by i.v. injection of *Listeria*-OVA produced specific CD8 T cells with more robust ability to perform cytotoxic killing. However, we were still able to detect significant OVA-specific T cells with killing capacity following oral gavage infection, mimicking the natural route of infection of the bacterium. Interestingly, transgenic mice showed increased susceptibility to infection compared to wild type C57BL/6 regardless of the route of administration and therefore required a 20-1000 fold reduction in the dose of bacteria used for infection. This may be due in part to the different background strain of the transgenic mice since the expression of the H-2K^b^ and ΔY H-2K^b^ mutant genes have been bred to C3H/He animals and particular strains of mice have been shown to have distinct differences in resistance or susceptibility to *Listeria* [35].

The novel findings that antigen-specific T cells are deficient in number and in function in ΔY mice following *Listeria* infection *in vivo* demonstrates that development of functional Ag-specific T cells is severely impaired following infection due to the MHC I cytoplasmic tail mutation and implies a dependence on MHC I targeting and cross-presentation of bacterial antigens for loading and subsequent priming of CD8 T cells. The failure of the CD8 Ag-specific T cells from ΔY mice to expand following infection could also be detected as a significantly lower total number of CD8 T cells recovered after infection. A model of early events in the T cell response to *Listeria* infection has been described whereby almost all peripheral T cells become partially activated within one day. This is followed by massive T cell depletion of nonspecific T cells while Ag-specific T cells begin to divide and become fully activated to become the majority of the peripheral T cells by days 7–9, at the peak of the response [31], [32]. In this context, we propose that the cross-presentation deficiency in ΔY mice results in T cells receiving inadequate activation signals which do not clonally expand and instead undergo apoptosis with the manifestation of a large reduction in CD8 T cell number at the peak of *Listeria* infection response. Further, ΔY DCs can only present endogenously. Therefore, if they become directly infected with *Listeria* then they would become targets for any CTL produced, contributing to the loss in CD8 T cell number.

Cross-presentation of many types of antigens has been reported [14]. In this work, we have used the MHC I cytoplasmic tail transgenic mice to dissect the importance of the mechanism of cross-presentation within the overall context of MHC I presentation following bacterial infection. We observed that dose-dependent T cell responses are activated following infection of K^b^WT and ΔY bmDCs, indicating that the endogenous route of processing is predominant following direct infection. Following cellular invasion, *Listeria* initially occupies an endosome/phagosome [36], [37] but upon acidification of the phagosome, LLO, a cholesterol-dependent pore-forming toxin, is secreted and blocks phagosome-lysosome fusion by generating small pores that uncouple pH and calcium gradients across the phagosome membrane [38]. This acts to destroy the phagosomal membrane and allows escape of bacteria into the cytosol where they can replicate. In this way, *Listeria*-derived proteins are expected to deliver predominantly to the endogenous route of processing. However, we also observed that DCs from ΔY mice showed significantly reduced ability to activate T cells following direct infection with *Listeria*-OVA *in vitro* indicating the contribution of cross-presentation, probably due to the presence of extracellular dead bacteria. In addition, DCs have been shown to have a special ability to alkalinize phagosomes through the activity of NADPH oxidase [39]. LLO-mediated escape of *Listeria* to the cytosol can be inhibited by alkalinization of the phagosome [37] and bacterial escape from vesicles is approximately 10-fold less active at neutral compared to acidic pH [40]. Thus, the pH of DC *Listeria*-containing phagosomes may influence the timing of bacterial escape and also contribute to the exogenous route of processing following direct infection with *Listeria*.

Our previous studies demonstrated that MHC I tyrosine-based motif is required for constitutive internalization of MHC I molecules from the cell surface into early endosomes and lysosomes where loading of exogenously-derived antigen can occur [6]. It is proposed that surface K^b^WT molecules form an abundant source of MHC I for endosomal and lysosomal peptide-loading of exogenously-derived bacterial antigens. We examined loading of *Listeria*-OVA-derived antigens on APCs following direct infection of APCs and staining of MHC I-OVA complexes and despite significant differences between ΔY and K^b^WT APCs to stimulate T cell responses, were unable to detect significant differences in processing and loading of *Listeria*-derived OVA antigen. However, significant differences in the level of surface H-2K^b^-OVA_(257–264)_ complexes could be detected following infection and peptide pulsing. Considering our previous results, we propose that following infection, MHC molecules internalizing from the surface do not sort to compartments compatible with cross-presentation for recycling to the surface, causing an overall decrease in the level of H-2K^b^-OVA_(257–264)_ complexes present. The equilibrium of surface levels of MHC I molecules is a balance between new synthesis and constitutive internalization for degradation or recycling. Therefore, we conclude that aberrant recycling of MHC I molecules on ΔY APCs following infection may contribute to the decreased overall levels of surface H-2K^b^-OVA_(257–264)_ complexes, the differences of which are only detectable following peptide pulsing.

**Figure 7:**
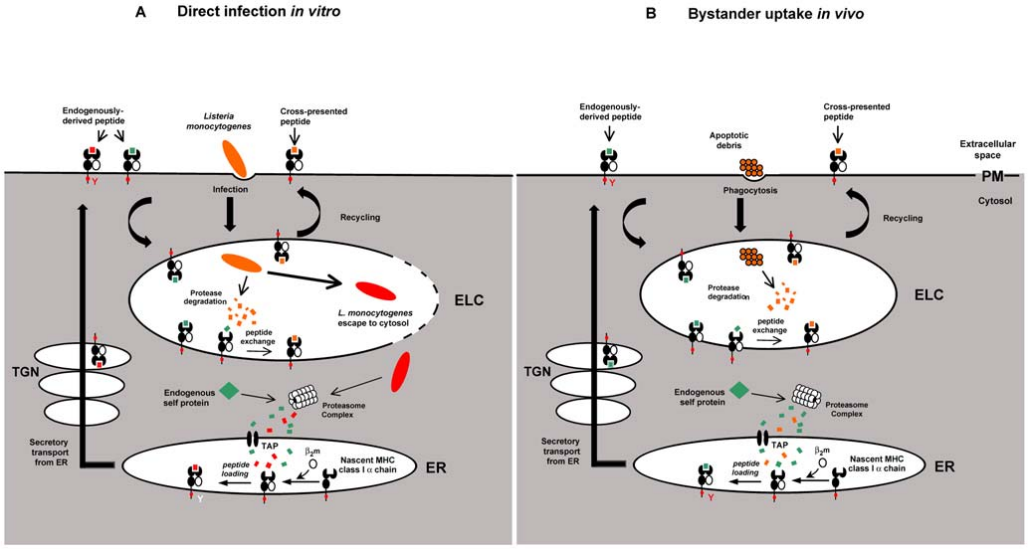
Proposed mechanisms of *Listeria*-derived antigen sampling following *in vitro* or *in vivo* infection. Schematic representation of the mechanisms involved in functional sampling of *Listeria*-derived antigens depending on the route of infection. A. *Listeria* directs its own phagocytic uptake into the cell. Within the phagosome, protease-mediated degradation of the bacterium generates peptides capable of binding MHC I molecules recycling from the surface, a process dependant on an intact tyrosine (Y) residue in the cytoplasmic tail of the MHC I molecule. Peptide exchange occurs allowing bacterially-derived antigens to be loaded on MHC I molecules and trafficked to the surface for cross-presentation. Predominantly, toxin-induced pore-forming of the phagosome occurs to allow the escape of the bacterium into the cytosol. Within the cytosol, *Listeria* is targeted for proteosome –mediated degradation and antigen transport to the ER where bacterial peptides are loaded on newly synthesized MHC I molecules. Loaded MHC I molecules follow the secretory pathway through the trans golgi network (TGN) to the surface for presentation. B. Following infection *in vivo*, directly infected cells undergo apoptosis, providing antigenic material for uptake by bystander DCs. In this scenario, apoptotic material containing bacterially-derived antigens is phagocytosed into a compartment competent for cross-presentation, named the endolysosomal compartment (ELC). MHC I molecules recycle from the surface into this compartment, peptide exchange occurs and bacterially-derived peptide antigens are loaded and recycled to the cell surface for cross-presentation.

Overall, the contribution of cross-presentation is likely to differ depending on mode of infection i.e. direct infection of APCs versus oral infection *in vivo*. Figure 7 describes mechanisms of cross-presentation that may occur depending on the route of infection. Protease-mediated degradation of the bacterium following direct infection *in vitro* within endolysosomal compartments competent for cross-presentation would provide peptides capable of binding recycling MHC I molecules. However, our data suggest that escape of the bacterium into the cytosol is predominant following infection *in vitro* whereby the bacterium is subsequently targeted for proteosome –mediated degradation and endogenous processing. Conversely, our results establish that, indeed cross-priming of CD8 T cells is an essential mechanism required to generate primary cellular immune responses following *in vivo* infection with *Listeria*. A mechanism for priming of CD8 T cells following bacterial oral infection across the gastrointestinal barrier is likely through bystander acquisition of bacterial antigen. We, and others have shown that *Listeria* infection causes substantial depletion of T cells by apoptosis. These cells may also be infected [41] necessitating bystander DCs to cross-present and prime CTL. Hereby, we propose that directly infected cells may undergo apoptosis *in vivo*, thereby providing antigenic material for uptake and processing in cross presenting endolysosomal compartments by bystander DCs. Indeed, cross-priming initiated by pathogen-induced apoptosis has been described following infection with viruses and bacteria [18]–[20], [42] suggesting that transfer of antigens from apoptotic vesicles to bystander APCs is a general mechanism of cross-priming of CD8 T cells in infectious disease.The rules of engagement for initiating and priming immune responses are now being written. These new edicts will eventually find their application in modulating autoimmune processes and together with novel molecular adjuvants, will promote the efficacy of emerging vaccines. The depth and breath of applying this knowledge is only beginning to be tapped.

## Materials and Methods

### Mouse strains and bacterial infections *in vivo*

C3H/He mice transgenic for the expression of wild-type MHC I allele H-2K^b^ (K^b^WT) or cytoplasmic tail tyrosine mutant (ΔY) were used in these studies as described previously [5]. C57BL/6 mice of H-2K^b^ haplotype were purchased from Charles River (St. Constant, QE, Canada). The transporter associated with antigen processing 1 knock out (TAP1−/−) and transgenic OT-I mice expressing a TCR specific for OVA_(257–264)_ peptide on a C57BL/6 background were purchased from Jackson Laboratory (Br Harbor, ME). Mice were maintained under specific pathogen-free conditions and fed the normal mouse diet ad libitum. All mouse protocols were approved and performed in accordance with the requirements set out by the Canadian Council on Animal Care.

A recombinant form of *Listeria monocytogenes* encoding Ovalbumin and an erythromycin-resistance marker was used for these studies (*Listeria*-OVA). An Antigen expression cassette consisting of the coding sequence of OVA fused to the signal sequence and promoter of the hly gene and an erythromycin resistance gene was introduced into *Listeria*, and double-crossed onto the *Listeria* chromosome by homologous recombination, as previously described [43].

Mice were infected with *Listeria*-OVA by oral gavage with 5e^6^-1e^9^ colony forming units (cfu) in 100 µl 0.1 M Hepes-PBS, as indicated or by i.v. with 1×10^4^ cfu in 100 µl PBS. Actual cfu were calculated following infection by plating dilutions of the inoculum. Mock oral infections were performed with 0.1 M Hepes-PBS. PBS alone was used for mock i.v. infections.

### Detection of CD8-specific T cell responses to *Listeria*-OVA

At the indicated times following infection, lymphocytes were isolated by Ficoll-paque (Amersham Biosciences) centrifugation and OVA-specific CD8 T cells were detected directly using a H-2K^b^/OVA_(257–264)_ tetramer (immunomics-BeckmanCouler^TM^). Otherwise, splenocytes were cultured for 5 days in RPMI 1640 medium (Sigma-Aldrich) supplemented with 10% FCS (HiClone), 100 U/ml penicillin, 100 µg/ml streptomycin (Invitrogen Life Technologies), nonessential amino acids (0.1 mM), sodium pyruvate (1 mM), 2-mercaptoethanol (50 muM), L-glutamine (2 mM) and 1 µM OVA_(257–264)_ (SIINFEKL) H-2K^b^-restricted peptide. Following *in vitro* boosting, CD8 T cells were tetramer stained and analysed by flow cytometry.

Cytotoxic T lymphocyte (CTL) assays were performed using a standard ^51^Chromimum (^51^Cr)-release assay. Splenocytes, following *in vitro* boosting with SIINFEKL peptide at 1 µM concentration for 5 days, were washed and used as effector cells. RMA-S incubated with 1 µM OVA_(257–264)_ and labeled with sodium chromate (100 µCi, Amersham) for 1 h were used as target cells. The CTL activity of activated CD8 T cells was assessed at various effector: target (E:T) ratios in a 4 h ^51^Cr-release assay at 37°C. Percent specific lysis was calculated as 100% x (cpm [experimental] - cpm [spontaneous release])/(cpm [maximum release] - cpm [spontaneous release]). All assays were performed in triplicate.

### Bone marrow-derived DC culture

Bone marrow-derived DCs (bmDCs) were prepared according to the procedure developed by Lutz et al. [44]. Cells flushed from femurs and tibias were plated in RPMI medium (Sigma-Aldrich) with 10% FBS (HiClone), 100 U/ml penicillin, 100 µg/ml streptomycin (Invitrogen Life Technologies), nonessential amino acids (0.1 mM), sodium pyruvate (1 mM), 2-mercaptoethanol (50 muM), L-glutamine (2 mM) and 1% GM-CSF containing supernatant from the X-63Ag8-GM-CSF-transfected cell line [45]. Cells were washed on days 3 and 6, 8, with culture media and 0.5% GM-CSF. DCs were routinely 95% CD11c^+^ and displayed low to intermediate levels of CD40, CD80, CD86 and MHC II, while expressing intermediate levels of MHC I, characteristic of immature DCs.

### Bacterial infection *in vitro*

bmDCs or splenocytes were harvested and plated in 48-well plates at a density of 5e^5^/well and infected with *Listeria*-OVA at 0.001-1 multiplicities of infection (MOI) for 4 h in antibiotic-free medium. After 4 h cells were washed twice in HBSS, resuspended in media with antibiotics (chloramphenicol, 10 µg/ml; Sigma), and infected cells were cultured for an additional 18 h. As positive controls for cross-presentation, bmDCs were incubated with soluble OVA at 10 and 30 mg/ml concentrations overnight. BmDCs were also incubated with 1 µM H-2K^b^-restricted OVA_(257–264)_ peptide for 1 h as positive control for B3Z assay.

### B3Z T cell activation assay

B3Z is a T cell hybridoma expressing a TCR that specifically recognizes OVA_(257–264)_ (SIINFEKL) in the context of H-2K^b^. The cells carry a beta-galactosidase (lacZ) construct driven by nuclear factor of activated T cells elements from the interleukin 2 promoter [46]. Processing and subsequent presentation of *Listeria*-derived OVA or soluble OVA was measured using Chlorophenol Red Galactopyranoside (CPRG, Calbiochem) as a chemiluminescent substrate for the detection of lacZ activity in B3Z lysates.

BmDCs were incubated with B3Z cells at 1∶1 ratio for 18 h. Individual cultures were lysed by addition of CPRG lysis buffer (100 mM 2-ME, 9 mM MgCl2, 0.125% Nonidet P-40, 0.15 mM chlorophenol red β-galactoside in PBS). Following 18 h incubation at room temperature, in the dark, absorption was read at 570 nm, with 650 nm as the reference wavelength. Uninfected bmDCs incubated with B3Z served as background controls. Fold induction index was calculated by dividing induced activity by background.

### OT-I T cell proliferation assay

Splenocytes were isolated from K^b^WT and ΔY transgenic mice and were infected with varying MOIs of *Listeria*-OVA for 4 h before the infection was terminated by washing cells with HBSS and resuspending cells in media with 10 µg/ml of chloramphenicol. Infected splenocytes were incubated overnight to allow processing and presentation of *Listeria*-derived antigens. Next day, splenocytes were cocultured at a 1∶1 ratio with OT-I T cells expressing a transgenic TCR that specifically recognizes H-2K^b^ in complex with OVA_(257–264)_ [47]. T cell proliferation was determined 48 h later by ^3^H-thymidine incorporation. Uninfected splenocytes incubated with OT-I T cells served as background controls and fold increase in proliferation was calculated by dividing induced activity by background.

### Detection of H-2K^b^OVA_(257–264)_ loaded complexes

Processing and loading of *Listeria*-OVA-derived antigens on MHC I was detected with a monoclonal antibody (mAb 25.D1.16) that specifically recognizes H-2K^b^-OVA_(257–264)_ complexes [48].

APCs were blocked anti-mouse Fc receptor (BD) and stained with biotin labeled antibody mAb 25.D1.16 followed by phycoerythrin-conjugated rat anti-mouse IgG1 (BD). Cells were then washed and analysed by flow cytometry. Uninfected APCs or infected APCs pulsed with 1 µM OVA_(257–264)_ peptide for 1 h were used as negative and positive controls, respectively.

### Statistical Analysis

A student's T test was used to compare numbers and percentages of CD4/CD8 or H-2K^b^OVA_(257–264)_ tetramer labeled cells from K^b^WT and ΔY transgenic mice. Statistical differences in B3Z T cell activation between K^b^WT, ΔY and TAP1−/− mice were also analyzed by student's T tests. Differences between two populations were considered statistically different if *p<0.05, **p<0.005 or ***p<0.0005.
